# High Connectivity of Animal Populations in Deep-Sea Hydrothermal Vent Fields in the Central Indian Ridge Relevant to Its Geological Setting

**DOI:** 10.1371/journal.pone.0081570

**Published:** 2013-12-16

**Authors:** Girish Beedessee, Hiromi Watanabe, Tomomi Ogura, Suguru Nemoto, Takuya Yahagi, Satoshi Nakagawa, Kentaro Nakamura, Ken Takai, Meera Koonjul, Daniel E. P. Marie

**Affiliations:** 1 Mauritius Oceanography Institute, Quatre-Bornes, Mauritius; 2 Institute of Biogeosciences, Japan Agency for Marine-Earth Science and Technology, Yokosuka, Kanagawa, Japan; 3 Graduate School of Marine Science and Technoloy, Tokyo University of Marine Science and Technology, Minato, Tokyo, Japan; 4 Enoshima Aquarium, Fujisawa, Kanagawa, Japan; 5 Atmosphere and Ocean Research Institute, the University of Tokyo, Kashiwa, Chiba, Japan; 6 Faculty of Fisheries Sciences, Hokkaido University, Hakodate, Hokkaido, Japan; 7 Precambrian Ecosystem Laboratory, Japan Agency for Marine-Earth Science and Technology, Yokosuka, Kanagawa, Japan; 8 Albion Fisheries Research Centre, Ministry of Fisheries, Petite Rivière, Mauritius; Dauphin Island Sea Lab, United States of America

## Abstract

Dispersal ability plays a key role in the maintenance of species in spatially and temporally discrete niches of deep-sea hydrothermal vent environments. On the basis of population genetic analyses in the eastern Pacific vent fields, dispersal of animals in the mid-oceanic ridge systems generally appears to be constrained by geographical barriers such as trenches, transform faults, and microplates. Four hydrothermal vent fields (the Kairei and Edmond fields near the Rodriguez Triple Junction, and the Dodo and Solitaire fields in the Central Indian Ridge) have been discovered in the mid-oceanic ridge system of the Indian Ocean. In the present study, we monitored the dispersal of four representative animals, *Austinograea rodriguezensis*, *Rimicaris kairei*, *Alviniconcha* and the scaly-foot gastropods, among these vent fields by using indirect methods, i.e., phylogenetic and population genetic analyses. For all four investigated species, we estimated potentially high connectivity, i.e., no genetic difference among the populations present in vent fields located several thousands of kilometers apart; however, the direction of migration appeared to differ among the species, probably because of different dispersal strategies. Comparison of the intermediate-spreading Central Indian Ridge with the fast-spreading East Pacific Rise and slow-spreading Mid-Atlantic Ridge revealed the presence of relatively high connectivity in the intermediate- and slow-spreading ridge systems. We propose that geological background, such as spreading rate which determines distance among vent fields, is related to the larval dispersal and population establishment of vent-endemic animal species, and may play an important role in controlling connectivity among populations within a biogeographical province.

## Introduction

More than 30 years have passed since the discovery of the first hydrothermal vents on the Galapagos Rift in the eastern Pacific Ocean [1]. Numerous vents continue to be found and catalogued along global mid-ocean ridge systems, back-arc spreading centers, and off-axis submarine volcanoes [2]. The vents are usually associated with dense assemblages of organisms, which are patchily distributed on the deep-sea floor [3], [4]. These communities are typically separated by tens to hundreds of kilometers along an actively spreading ridge, and by even greater distances between ridge segments. The existence of such communities highlights the significant contribution to geologically produced energy sources to chemosynthetic biomass production, and also the remarkable adaptability of life in deep-sea hydrothermal vent ecosystems [5].

Considering the vast geographical distribution of the hydrothermal vent sites [6], the animals inhabiting them are likely to possess exceptional colonization abilities, including high rates of dispersal, growth, and reproduction [7], [8], [9]; further, dispersal probably occurs mainly via the planktonic larval or juvenile stages [10]. Dispersal and subsequent reproduction results in a decrease in genetic differences among populations and an increase in the genetic variability within populations [11]. Negatively buoyant larvae mainly move with bottom currents in line with the axial valley of the ridge system [12], [13], [14], [15], while positively buoyant larvae can disperse more than several tens of meters above the seafloor in hydrothermal plumes [16]. Colonization is not a simple process, because dispersal is affected by several factors, particularly plate tectonics, which have contributed to the current biogeographical patterns of faunal diversity in vents around the globe [17], [18]. Dispersers may never reach a suitable habitat or may suffer from high mortality during transition. On the other hand, dispersal may allow them to explore new favorable habitat [19].

**Figure 1:**
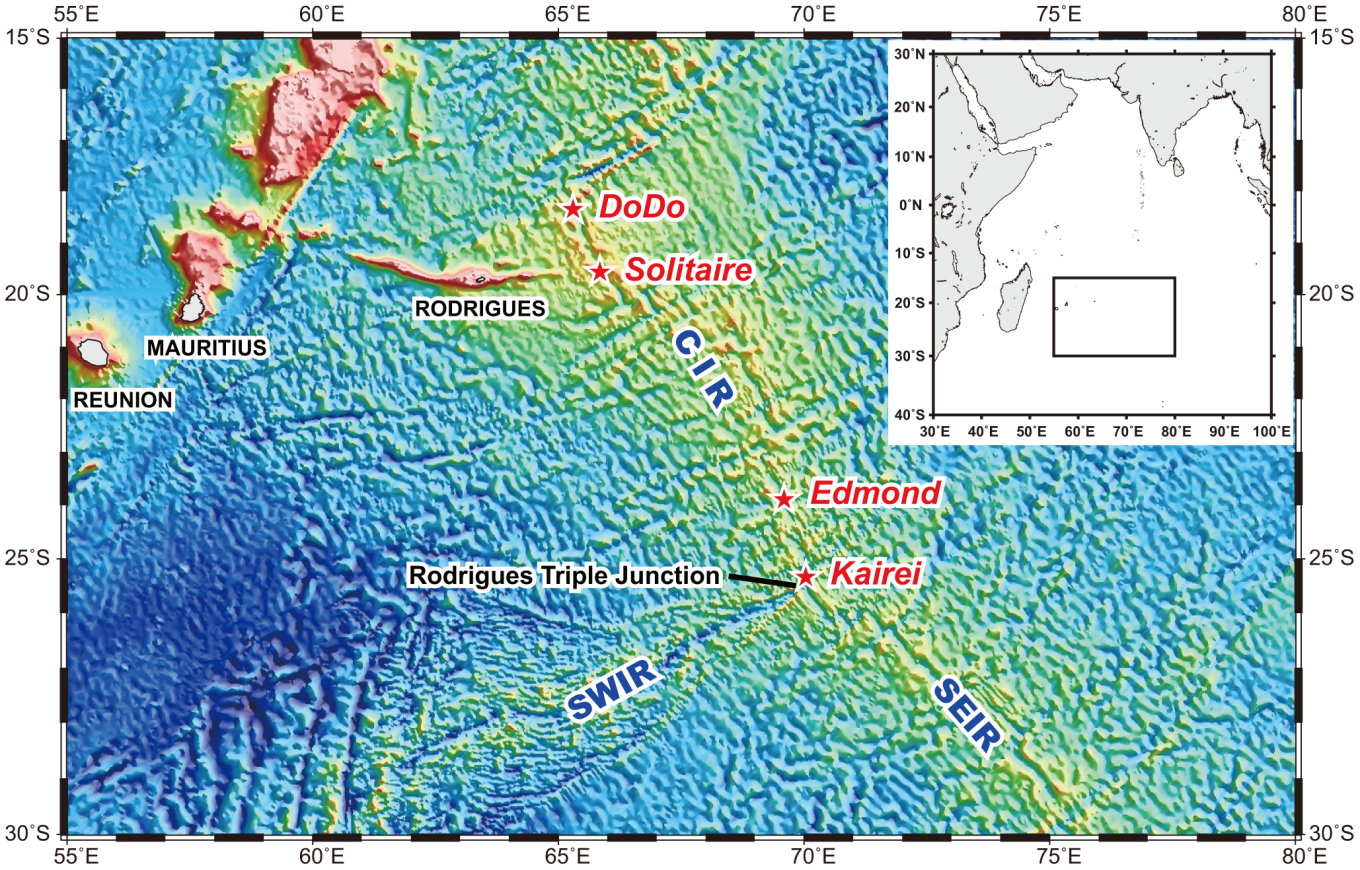
Location of the collection sites.

**Figure 2:**
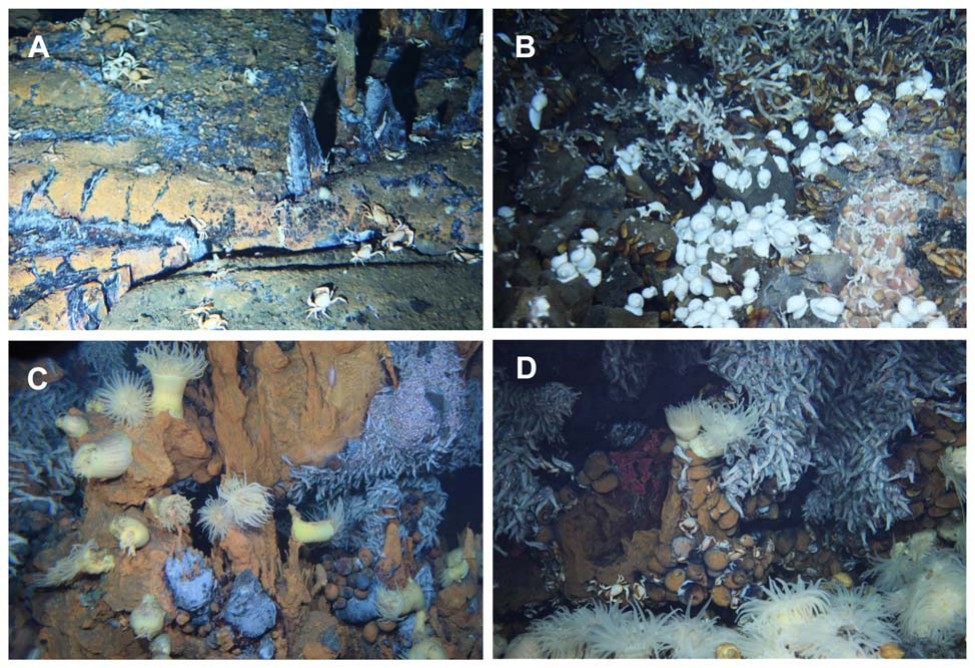
Photographs of the collection sites. A, Dodo site; B, Solitaire site; C, Edmond site; and D, Kairei site.

The dispersal, isolation, and speciation of vent endemic species constitute one of the key questions of deep-sea biology, particularly in deep-sea hydrothermal vents [9]. Several field-based studies have been conducted in different geographical provinces [17], [18], [20]. In the present study, we aimed to address these phenomena, with respect to hydrothermal systems, the Kairei and Edmond vent fields [21], [22], and two recently discovered vent fields, namely, the Dodo and Solitaire fields [23], located in segments 16 and 15, respectively, of the Central Indian Ridge (CIR) (Figures 1 and 2). Biogeographical features appear to be influenced by the timing and spacing of hydrothermal activities in a spreading axis [5]. The factors generally considered when elucidating the relationships of biogeography are spreading rate and spacing. Previous studies have indicated the presence of one active vent site per 100–350 km in the Mid-Atlantic Ridge (MAR) and one active vent site per 5 km in the East Pacific Rise (EPR) [5]. However, few investigations of the CIR have been conducted, and the spacing of vent fields in this axis remains unclear. The CIR has an intermediate spreading rate (50–60 mm/year), while the EPR and MAR have fast (180 mm/year) and slow (25 mm/year) spreading rates, respectively [5], [20]. These differences in spreading rates are expected to influence the biogeographical pattern, e.g., the distance between vent fields represents a greater biogeographical barrier in a slow-spreading axis than in a fast-spreading axis [5]. However, the results of recent analyses on gene flow contradict this hypothesis. On the basis of genetic analyses of deep-sea vent species in the EPR, some dispersal barriers such as faults, fracture zones, and intercalation of microplate and topographic depressions have been proposed [9]. Further, in the hydrothermal vent fields of the MAR, no genetic difference has been detected among populations of *Rimicaris exoculata* [24] or other animals [25]. Elucidation of the biogeographical patterns of the Indian Ocean hydrothermal vents will provide an insight into the relationships between geological background such as spreading rate, and larval dispersal or connectivity of vent animals.

In the present study, we investigated genetic diversity and population differentiation of four dominant species in the four hydrothermal vent fields along the CIR to discuss larval dispersal ability indirectly. In addition, we measured the size of the animals constituting each population, because this reflects many aspects of the life-history trait, and also variations among categories such as taxa and population [26]. In order to elucidate the potential effects of geological setting and geographical distance on dispersal, connectivity of the four vent species were compared to those observed in EPR and MAR, and discuss potential dispersal barriers encountered by animal populations associated with deep-sea hydrothermal vents.

## Materials and Methods

### Sample collection

The samples were collected in the four deep-sea hydrothermal vent fields in the CIR (Dodo, Solitaire, Edmond and Kairei fields). Among them, the Dodo and Solitaire fields are located in the Exclusive Economic Zone of Mauritius, and we conducted these researches with the permission of the Mauritius Prime Minister's Office, during the YK09-13 cruise (October to November 2009), by using the Human Observing Vehicle (HOV) *Shinkai6500* and R/V *Yokosuka* of the Japan Agency for Marine-Earth Science and Technology (JAMSTEC). We collected four representative animal species of the CIR vent fields, *Austinograea rodriguezensis* Tsuchida & Hashimoto 2002, *Rimicaris kairei* Watabe & Hashimoto 2002, *Alviniconcha* sp. type 3 (refer to [27]), and the scaly-foot gastropod from the hydrothermal vent fields shown in Figure 1. The geological background, geochemical characteristics, and species composition of the associated fauna were reported previously [27]. Immediately after recovery of HOV Shinkai6500, the specimens were stored in either 10% seawater-buffered formalin or 99.5% ethanol, or at −80°C (Table 1).

**Table 1:** Number of specimens used in the present study.

	No. of specimens used			
Site	*A. rodriguezensis*	*R. kairei*	*Alviniconcha* sp.	scaly-foot gastropod [23]
Dodo	10 (ND)	6 (ND)	-	-
Solitaire	20 (18)	40 (18)	17 (12)	ND (14)
Edmond	ND (ND)	373 (18)	ND (19)	-
Kairei	20 (18)	383 (18)	15 (13)	ND (19)

ND: no data in the present study, -: not observed. Numbers in parentheses are those used for population genetic analyses.

### Size distribution

With the exception of *Alviniconcha* sp. type 3, all of the collected specimens were used in size distribution analysis (Accidentally, only some of the *Alviniconcha* sp. type 3 specimens were measured.). The sizes of all individuals of the representative species in the four hydrothermal vent sites were measured. We examined differences in the average sizes of each local population by using non-parametric tests (Mann-Whitney U-test or Kruskal-Wallis test, depending on the number of populations), based on the following measurements: carapace width (CW) for *A. rodriguezensis*; carapace length (CL) for *R. kairei*; and shell width (SW) for *Alviniconcha* sp. type 3. Measurements for the scaly-foot gastropod were reported previously [23].

### DNA extraction, amplification, and sequencing

Genomic DNA was extracted from ethanol-fixed and frozen samples by using the DNeasy Tissue Extraction Kit (QIAGEN, Hilden, Germany). The extracted DNA of molluscs was treated with GeneReleaser (BioVenture, Marfreesboro, USA) before Polymerase Chain Reaction (PCR). The target DNA sequence of the present study was a mitochondrial gene, cytochrome oxidase *c* subunit 1 (COI). For *R. kairei*, a 535-bp fragment of COI was amplified by using the primers LCO1490 and HCO2198 [28]. For *A. rodriguezensis*, an 810-bp fragment was amplified by using the primers LCO1490 and COI-6 [29]. For, *Alviniconcha* sp. type 3, a 491-bp fragment was amplified by using the primers COI-B [30] and COI-6. The reactions were performed in 20-µL reaction mixtures containing genomic DNA, *ExTaq* buffer, 0.3 mM of dNTP mix, 1 mM of each primer, and 0.75 units of ExTaq DNA polymerase (TaKaRa Bio, Ohtsu, Japan). The cycling parameters were 94°C for 2 min, 30 cycles of 94°C for 30 s, 50°C for 30 s, 72°C for 1 min, and a final elongation step at 72°C for 2 min. The PCR products were purified by using ExoSAP-IT® (USB®, Affymax, Santa Clara, USA), and sequenced bidirectionally by using the same primers as those described above for PCR, and the BigDye® Terminator Cycle Sequencing Kit Version 3.1 (Applied Biosystems®, Life Technologies Corporation, Carlsbad, USA). The products were purified by using a Gel Filtration Cartridge (Edge BioSystems, Gaithersburg, USA) or BigDye XTerminator® Kit (Applied Biosystems®) treatment, before sequencing analyses by using ABI 3130 or ABI 3730 DNA sequencers (Applied Biosystems®).

### Molecular data analysis

The obtained forward and reverse sequences were assembled into contigs by using the program ATGC (Genetyx® Version 6, Genetyx, Tokyo, Japan), and aligned by eye. All of the detected haplotypes were used for phylogenetic analyses in neighbor-joining (NJ) and maximum likelihood (ML) algorithms. The phylogeny of each animal was clarified by using the software package MEGA 5 [31]. The molecular phylogenetic tree was reconstructed with previously published data in DNA databanks (NCBI, DDBJ, and EMBL; accession numbers are shown in the phylogenetic trees). Additionally, all of the obtained sequences were used for population-level analyses. The parsimonious haplotype network of each species was estimated by using the software package TCS 1.21 [32]. For each population, we estimated genetic diversity indices (*H*?: gene diversity, *π*: nucleotide diversity, and mismatch distribution), and conducted statistical tests for genetic structure, by using Arlequin ver. 3.11 [33]. In mismatch distribution analysis, we applied the goodness-of-fit test to compare the observed mismatch distribution, with the mismatch distribution obtained by using the sudden expansion model. For those species with more than three populations (*R. kairei* and *Alviniconcha* sp. type 3), we used analysis of molecular variance (AMOVA) to examine the genetic structure between northern segment populations (Dodo and Solitaire fields), and populations near the triple junction (Edmond and Kairei fields). The relative number of migrants per generation was estimated by using MIGRATE ver. 3.2.1 [34]. A part of the population-level analyses included the previously reported sequences for the scaly-foot gastropod [23].

## Results

### Size distribution

**Figure 3:**
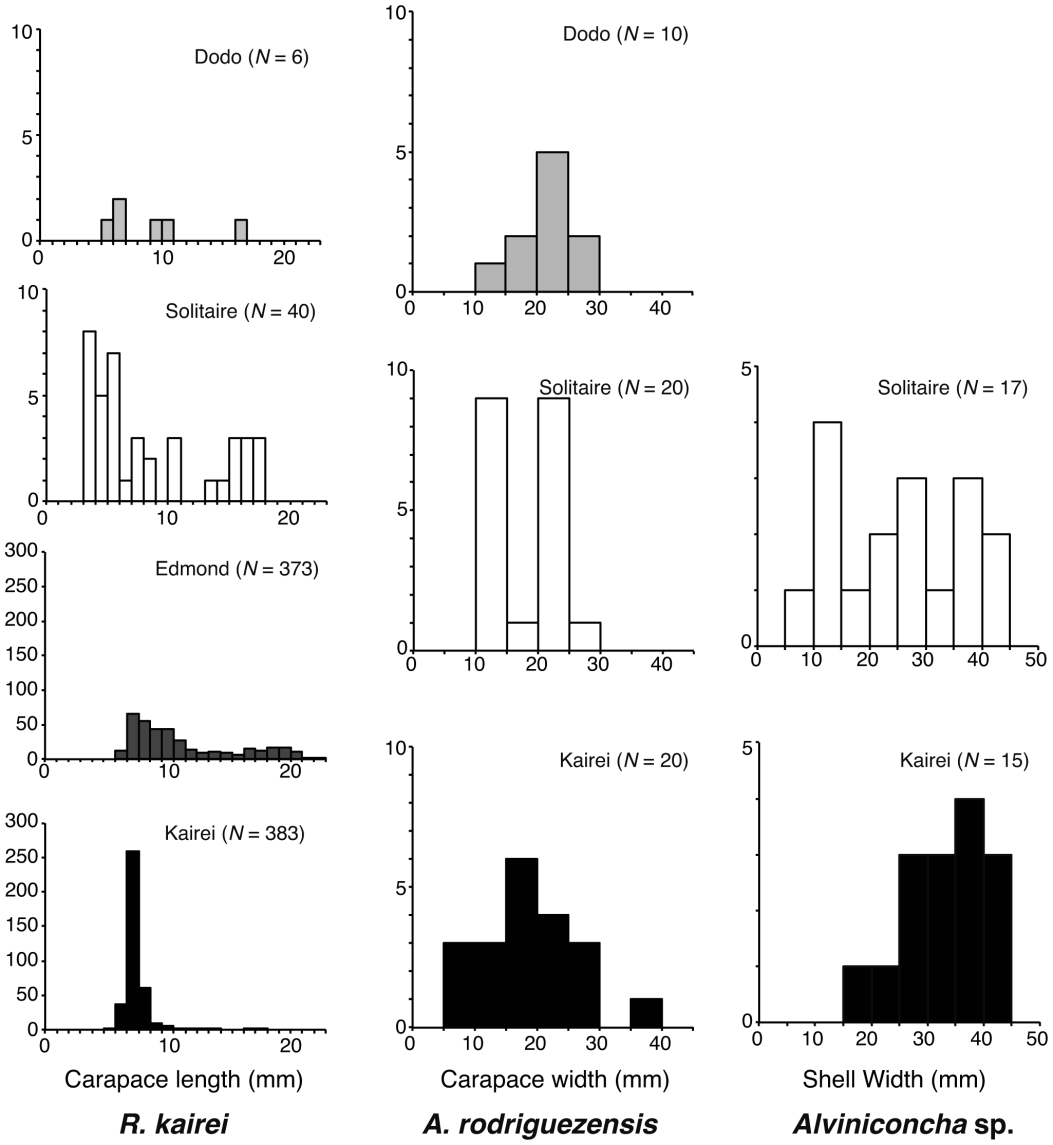
Size histograms of the four representative species in the CIR. A, *Austinograea rodriguezensis*; B, *Rimicaris kairei*; and C, *Alviniconcha* sp. type 3.

The obtained size distributions of the four representative species are summarized in Figure 3. The results for the scaly-foot gastropod were reported previously [23].

*A. rodriguezensis* was observed in all four vent fields of the Indian Ocean. The Dodo population had the largest CW (25.19±5.00 mm; average ± standard deviation), while the Solitaire population had the smallest CW (20.99±4.79 mm). The Kairei population showed an intermediate CW (22.90±7.61 mm), but only a single peak in size distribution (Figure 3A). The Kruskal-Wallis test revealed no significant difference among the CW values of local *A. rodriguezensis* populations (*H* = 3.848, *P* = 0.146).

*R. kairei* was dominant in the vent fields near the Rodriguez Triple Junction (Edmond and Kairei fields), but sparse in the northern segments (Dodo and Solitaire fields; Figure 2). Therefore, fewer individuals were collected from the Dodo and Solitaire fields (6 and 40, respectively) than from the Edmond and Kairei fields (373 and 383, respectively). The Edmond population had the largest CL (11.61±4.43 mm), while the Kairei population had the smallest CL (7.70±1.39 mm; Figure 3B). A single prominent peak in size distribution was present in the Kairei population; however, other populations showed multiple peaks or gently sloping size distributions (CL: Dodo population, 10.10±4.12 mm; Solitaire population, 9.41±5.06 mm). The Kruskal-Wallis test revealed a significant difference among the CL values of local *R. kairei* populations (*H* = 260.3, *P*<0.001).

*Alviniconcha* sp. type 3 was not observed in the Dodo field but was present in the remaining three fields. The Kairei population had a larger SW (37.89±7.48 mm) than did the Solitaire population (29.82±11.66 mm; Figure 3C). A single prominent peak in size distribution was present in the Kairei population; however, the Solitaire population showed multiple peaks. Mann-Whitney's *U*-test revealed no significant difference between the SW values of the Kairei and Solitaire populations (*U* = 78.50, *P* = 0.0651).

### Molecular phylogenetic analyses

**Figure 4:**
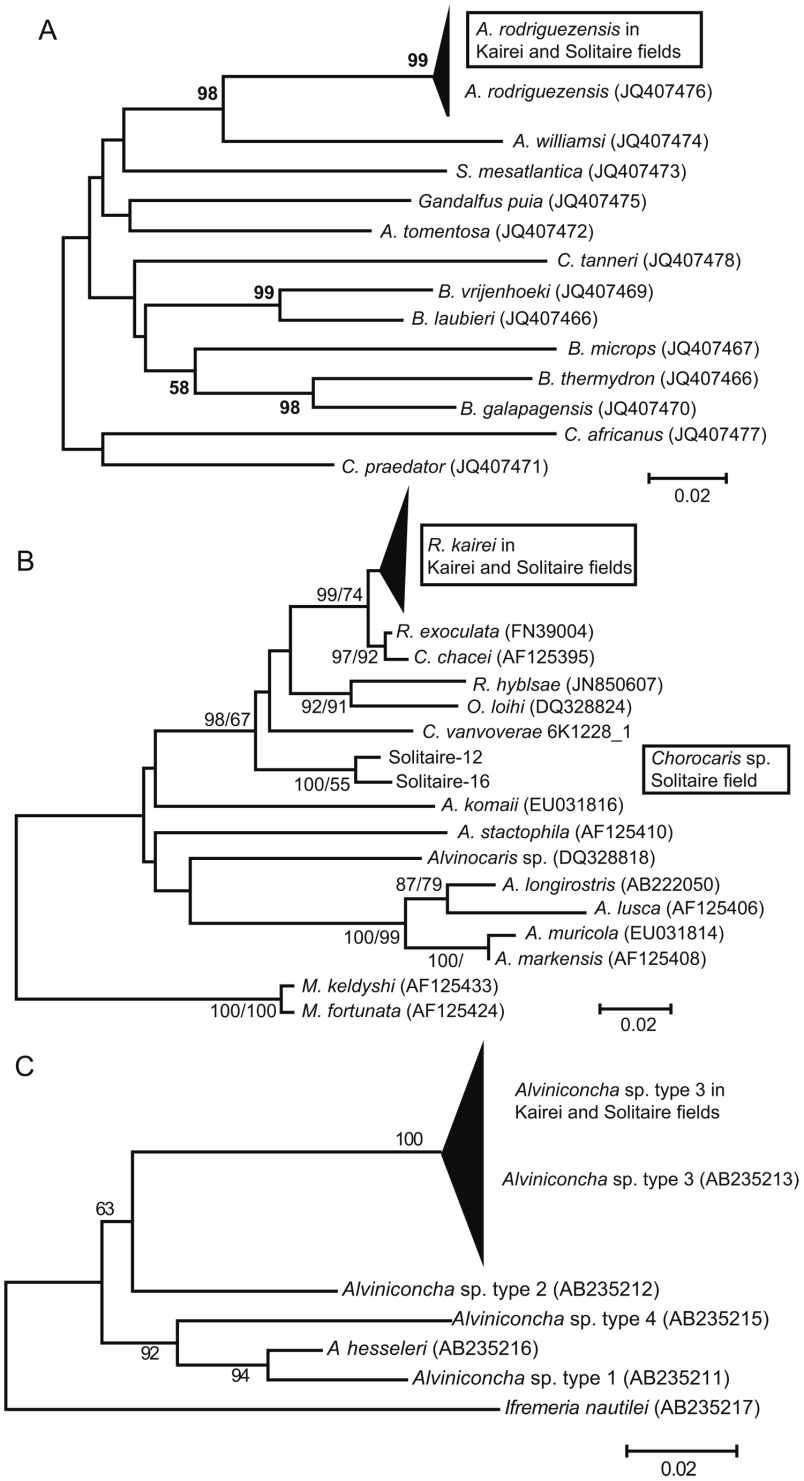
Molecular phylogenetic trees of the four representative species in the CIR. A, *A. rodriguezensis*; B, *R. kairei*; and C, *Alviniconcha* sp. type 3.

In total 167 COI sequences (78 haplotypic sequences were registered as AB817031–AB813148 in DDBJ, and also in NCBI and EMBL; 33 sequences for the scaly-foot gastropod were reported in [23]) were obtained. All of the individuals collected and analyzed comprised a single lineage in each species. The results of phylogenetic analyses revealed that the haplotype lineages of *A. rodriguezensis* and *Alviniconcha* sp. type 3 were specific to the CIR, with high bootstrapping values (99 and 100; Figure 4A, C). On the other hand, the lineage of *R. kairei* in the CIR was supported by a bootstrapping value of <50 (Figure 4B).

### Genetic diversity and population structure analyses

**Figure 5:**
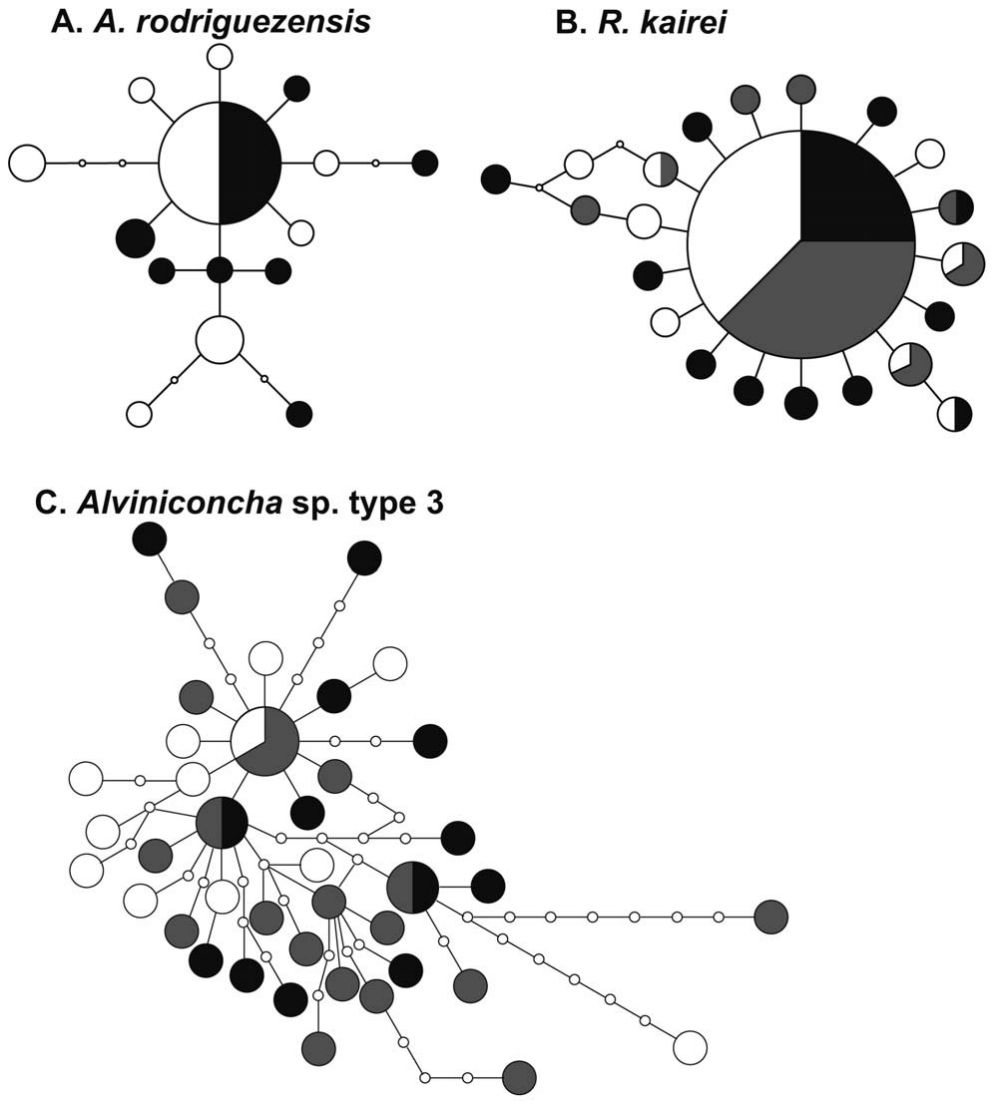
Haplotype networks of the four representative species in the CIR. A, *A. rodriguezensis*; B, *R. kairei*; and C: *Alviniconcha* sp. type 3. Black, population in the Kairei field; gray, population in the Edmond site; and white, population in the Solitaire field.

**Figure 6:**
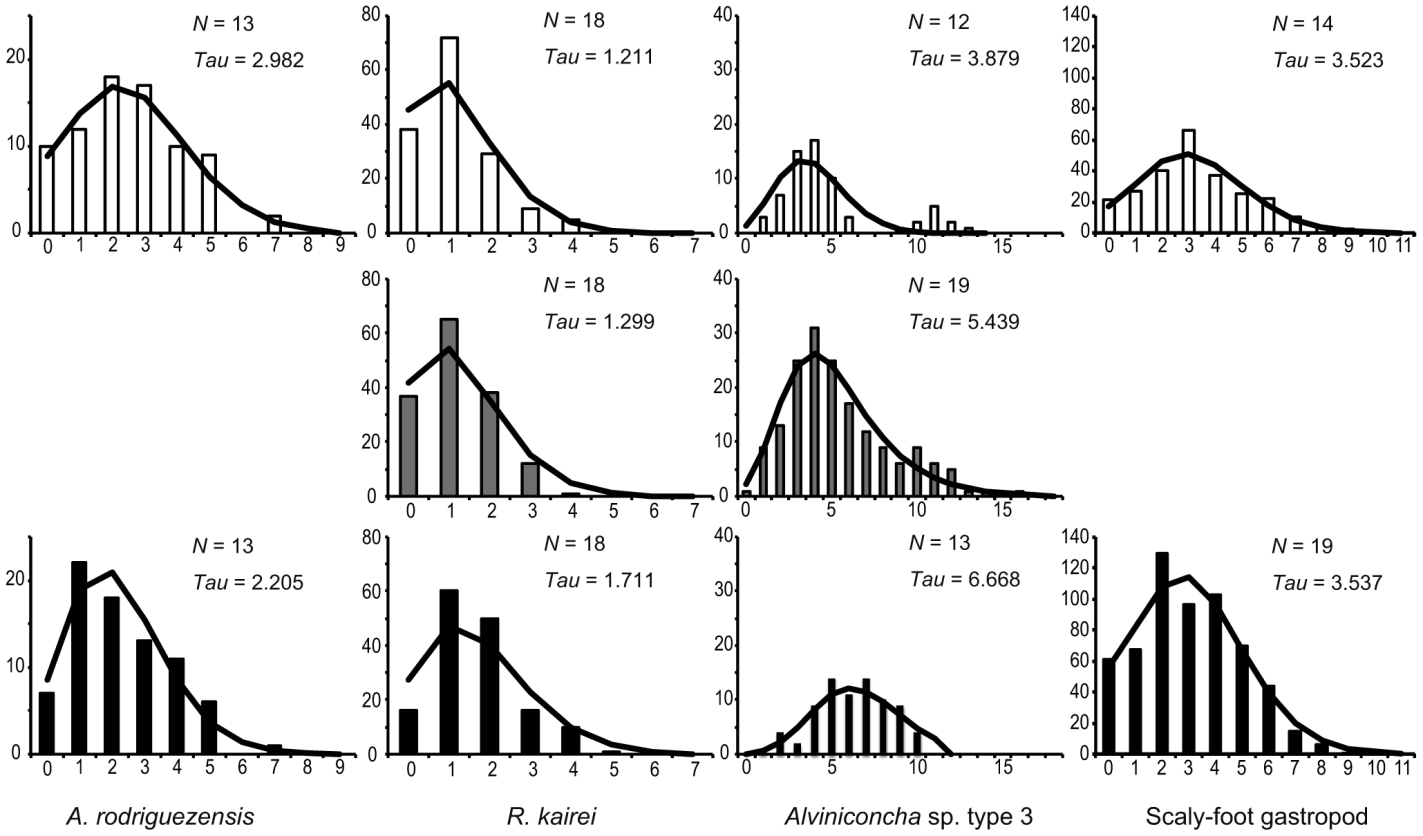
Mismatch distribution of the four representative species in the CIR. A, *A. rodriguezensis*; B, *R. kairei*; C, *Alviniconcha* sp. type 3; and D, scaly-foot gastropod. Black, population in the Kairei field; gray, population in the Edmond site; and white, population in the Solitaire field.

We reconstructed and estimated haplotype networks (Figure 5) and genetic diversity indices (Tables 2 and 3), including mismatch distribution (Figure 6), for the four representative animals from the CIR vent fields. In addition, we calculated pairwise *F_ST_* and Wright's exact tests for each population of the four species (Tables 4, 5, 6 and 7), and conducted AMOVA for *R. kairei* and *Alviniconcha* sp. type 3 (Tables 8 and 9).

**Table 2:** Haplotype diversities of the populations of the representative species of Indian Ocean hydrothermal vent fields.

	*A. rodriguezensis*	*R. kairei*	*Alviniconcha* sp.	scaly-foot gastropod
Solitaire	0.8718±0.0670	0.7516±0.1031	1.0000±0.0340	0.9170±0.0419
Edmond		0.7582±0.1056	0.9942±0.0193	
Kairei	0.9103±0.0683	0.8954±0.0653	1.0000±0.0302	0.8975±0.0443

**Table 3:** Nucleotide diversities of the populations of the representative species of Indian Ocean hydrothermal vent fields.

	*A. rodriguezensis*	*R. kairei*	*Alviniconcha* sp.	scaly-foot gastropod
Solitaire	0.003138±0.002026	0.002162±0.001636	0.004568±0.002700	0.005602±0.003341
Edmond		0.002211±0.001663	0.005140±0.002894	
Kairei	0.002817±0.001856	0.003091±0.002132	0.005889±0.003362	0.005236±0.003107

**Table 4:** Results of genetic structure analyses on *A. rodriguezensis*.

	Kairei	Solitaire
**Kairei**		NS
**Solitaire**	0.00407	

*F_ST_*, upper right: Wright's Exact test. NS: not significant in 5% level. Lower left: pairwise

**Table 5:** Results of genetic structure analyses on *R. kairei*.

	Kairei	Edmond	Solitaire
**Kairei**		NS	NS
**Edmond**	−0.00774		NS
**Solitaire**	0.00591	−0.00814	

*F_ST_*, upper right: Wright's Exact test. NS: not significant in 5% level. Lower left: pairwise

**Table 6:** Results of genetic structure analyses on *Alviniconcha* sp. type 3.

	Kairei	Edmond	Solitaire
**Kairei**		NS	NS
**Edmond**	0.0044		NS
**Solitaire**	0.01394	0.06738**	

*F_ST_*, upper right: Wright's Exact test. NS: not significant in 5% level. Lower left: pairwise

**Table 7:** Results of genetic structure analyses on scaly-foot gastropod.

	Kairei	Solitaire
**Kairei**		NS
**Solitaire**	−0.00957	

*F_ST_*, upper right: Wright's Exact test. NS: not significant in 5% level. Lower left: pairwise

**Table 8:** Results of AMOVA on *R. kairei*.

Source of Variation	d.f.	Sum of squares	Variance components	Percentage of variation
Among groups	1	0.648	0.00154	0.23
Among populations within groups	1	0.611	−0.00303	−0.46
Within populations	51	33.944	0.66558	100.22

**Table 9:** Results of AMOVA on *Alviniconcha* sp. type 3.

Source of Variation	d.f.	Sum of squares	Variance components	Percentage of variation
Among groups	1	4.806	0.10181	3.54
Among populations within groups	1	3.074	0.02074	0.72
Within populations	41	112.915	2.75403	95.74

**Figure 7:**
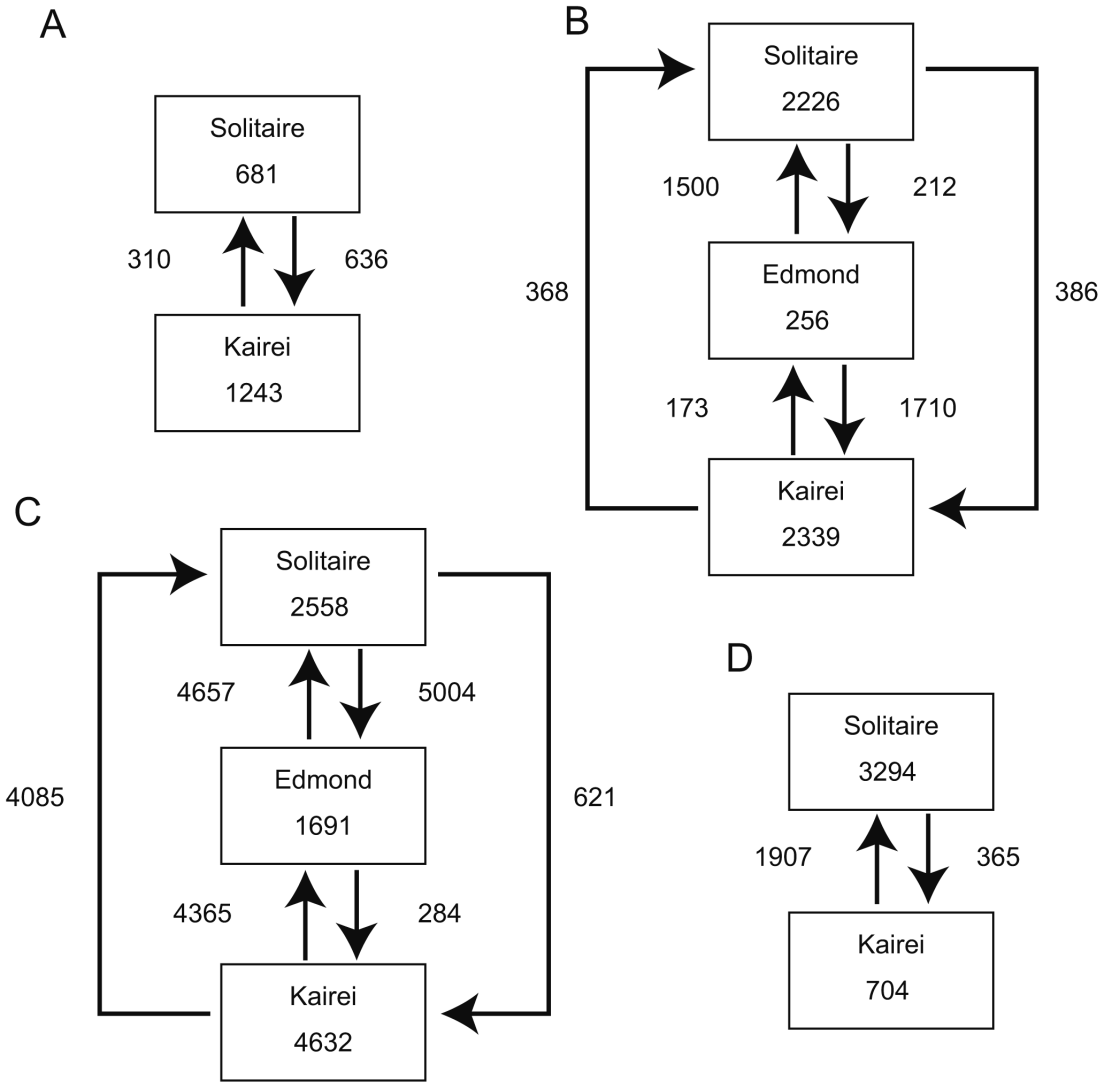
Schematic images of the results of MIGRATE analyses. A, *A. rodriguezensis*; B, *R. kairei*; C, *Alviniconcha* sp. type 3; and D, scaly-foot gastropod.

*A. rodriguezensis* showed the simple network and low genetic diversity among the four investigated species. Genetic diversity was higher in the Solitaire population than in the Kairei population. Pairwise *F_ST_* and Wright's exact tests indicated that the populations in the two CIR vent fields were not divided genetically (Table 4). The goodness-of-fit test revealed no significant difference between the observed and model frequencies (Solitaire population, *P* = 0.86; Kairei population, *P* = 0.55); further, sudden population expansion was not rejected. Migration analyses showed that the relative number of migrants per generation was about twice of those from the Solitaire population to the Kairei population than from the Kairei population to the Solitaire population (Figure 7A).

The haplotype network for *R. kairei* was estimated as a simple star-like network (Figure 5). Each of the estimated genetic diversity indices was the lowest among the four investigated species (Tables 2 and 3). Mismatch distribution of each population showed a single peak that did not differ significantly from the model frequency (goodness-of-fit test: Solitaire population, *P* = 0.20; Edmond population, *P* = 0.41; Kairei population, *P* = 0.14); further, sudden population expansion was not rejected. Pairwise *F_ST_* and Wright's exact tests revealed no significant difference among the examined populations (Table 5), even between the northern population (Solitaire) and the near-triple junction populations (Edmond and Kairei) (Table 8). The estimated number of migrants per generation was lower for all migrations directed toward the Kairei population (284 from the Edmond population; 621 from the Solitaire population) than for any other migration direction (>4085; Figure 7).

In comparison with the other investigated species, *Alviniconcha* sp. type 3 showed a more highly diversified haplotype network, i.e., a larger number of haplotypes constituting a more complicated network (Figure 5). The genetic diversity indices were high in the Kairei population and low in the Solitaire population (Tables 2 and 3, and Figure 6). The goodness-of-fit test revealed no significant difference between the observed and model frequencies (Solitaire population, *P* = 0.25; Edmond population, *P* = 0.78; Kairei population, *P* = 0.65); however, mismatch peaks were observed in mismatch numbers 10 and 11 for the Edmond and Solitaire populations, respectively. With the exception of the pairwise *F_ST_* test between the Solitaire and Edmond populations (Table 6), pairwise *F_ST_* and Wright's exact test revealed no significant difference among the examined populations. AMOVA between the northern population and the near-triple junction populations revealed no significant differences (Table 9). The relative number of migrants per generation was notably high for migrations from the Edmond population (1500 to the Solitaire population; 1710 to the Kairei population; Figure 7) than for any other migration direction (<500).

The genetic diversity indices for the scaly-foot gastropod were relatively high and comparable with those of *Alviniconcha* sp. type 3 (Tables 2 and 3). The goodness-of-fit test showed a single peak that did not differ significantly between the observed and model frequencies (Solitaire population, *P* = 0.49; Kairei population, *P* = 0.76; Figure 6); further, sudden expansion was not rejected. Pairwise *F_ST_* and Wright's exact tests revealed no genetic difference between the two populations (Table 7). The estimated number of migrants per generation was higher from the Kairei population to the Solitaire population (1907) than from the Solitaire population to the Kairei population (365; Figure 7).

## Discussion

The results of our present study indicate for the first time to examine population differentiation in the four representative species (*A. rodriguezensis*, *R. kairei*, *Alviniconcha* sp. type 3 and scaly-foot gastropod) in the four hydrothermal vent communities (Dodo, Solitaire, Edmond, and Kairei) along the CIR.

The vent crab *A. rodriguezensis* is distributed in all 4 hydrothermal vents at CIR and appears to be an important predator in the area, while not dominating the chemosynthetic communities in the region [35]. The size distribution pattern of *A. rodriguezensis* in the Solitaire population showed two peaks, rather than a single peak as observed for the Dodo and Kairei populations (Figure 3). The result indicates that two cohorts may exist in the Solitaire population, while the other two populations only consisted of a single cohort. The different number of cohort of the Solitaire population may reflect the difference in the colonization process in the Solitaire field, for example, those resulted from different reproductive period or growth rate. Differences in environmental cues were previously reported to cause variations in reproductive periodicity among hydrothermal vent crabs belonging to the genus *Bythograea* [36]. Reproductive features are known sometimes to differ among populations of the same species [26]. The environmental characteristics of the four hydrothermal vents differ in many aspects, as indicated by the physical and chemical characteristics of high-temperature end member (-like) hydrothermal fluids (Table 10). The results of our present study may indicate differing reproductive periods among populations of a single species in CIR. To increase the reliability of our results, the number of individuals should be increased. Our present genetic analyses did not include individuals from the Dodo and Edmond populations; however, we detected no significant difference in genetic diversity between the Solitaire and Kairei populations (Tables 4–7). This finding is consistent with the results of previous investigation of *A. rodriguezensis* populations in the Kairei and Edmond fields, which showed that the genetic diversity differed by only 0.2% in mitochondrial cytochrome *b* [22]; taken together, these findings suggest the existence of gene flow among the populations in CIR vent fields. In the present study, the results of mismatch distribution and other genetic diversity indices indicate that the Solitaire population has a higher genetic diversity than does the Kairei population. The high genetic diversity and broad size range of the *A. rodriguezensis* population in the Solitaire field suggest that this field represents the potential source population for the four investigated populations (Figure 7). Larvae of *Bythograea thermydron* have developed eyes and show phototactic behavior [37]. Therefore larvae of *A. rodriguezensis*, which probably use the surface current as a means of dispersal, are able to disperse among the four hydrothermal vent fields, which are separated by several transform faults and non-transform offsets.

**Table 10:** Characteristics of hydrothermal fluid in the four vent fields in CIR.

Site	Temperature (°C)	pH	H_2_ (mmol/L)	CH_4_ (mmol/kg)	CO_2_ (mmol/kg)	Fe (mmol/kg)	Chlorinity
**Dodo ** [**23**]	356	3.2	>2	∼0.02	∼4		brine-rich
**Solitaire ** [**23**]	296	4.8	0.46	∼0.05	∼8		vapor-rich
**Edmond ** [**22**]	382	∼3	0.2	0.4		∼14	brine-rich
**Kairei ** [**22**]	365	∼3	8.5	0.2		∼5	brine-rich

The vent shrimp, *R. kairei*, was the dominant species in all four hydrothermal vent fields along the CIR. The size distribution pattern differed among the populations, the Kairei population showed a single prominent peak, whereas the other populations showed broad and multiple peaks (Figure 3). Similar to *A. rodriguezensis*, differences in size distribution among the *R. kairei* populations may reflect differences in reproductive features. The Edmond population showed the broadest size distribution (Figure 3), and therefore probably had the largest number of individuals. The results of phylogenetic analyses revealed no clear endemicity of this shrimp in the Indian Ocean; in other words, *R. kairei* belongs to the same lineage as *R. exoculata* in the hydrothermal vent fields of the MAR. However, *R. kairei* differs morphologically from *R. exoculata* in the MAR [38]. The results of AMOVA analysis further revealed no significant genetic differences between the four examined *R. kairei* populations (Tables 5 and 8). This finding is consistent with that of a previous study, which showed no genetic differences between the Kairei and Edmond populations of swarming shrimps [22]. Vent shrimps in the family Alvinocarididae are believed to have long larval dispersal periods, during which they consume photosynthetic-based nutrition in the euphotic zone [39], [40]. Further, some larvae of vent shrimps have potential to survive for long periods at a distance of up to 100 km from the vent sites [41], [42]. On the other hand, the results of migration analyses estimated a biased number of migrants per generation among three populations, i.e., prominent exportation from the Edmond population to the Solitaire and Kairei populations (Figure 7). Among the four vent field populations, *R. kairei* was less abundant in the two northern vent populations (Dodo and Solitaire) than in the two vent populations near the Rodriguez Triple Junction (Kairei and Edmond) (Figures 2 and 3). Taken together, our results suggest that the Edmond field is the largest, and provides a larval supply for the other vent field populations along the CIR.

The large hairy gastropod, *Alviniconcha* sp. type 3, was present in the Solitaire, Edmond and Kairei fields. The size distribution pattern differed between the two fields; a single peak was present in the Kairei population and the broad-ranged population in the Solitaire field (Figure 3), implying that the population conditions were influenced by the different environments of the two vent fields (Table 10). In accordance with a previous study of Kairei field samples [25], the results of phylogenetic analyses revealed the specificity of *Alviniconcha* sp. type 3 to the CIR region. Further, in accordance with a previous study of the Kairei and Edmond populations based on the mitochondrial 16SrRNA sequence [22], we revealed no significant genetic differences among the three examined populations (Tables 6 and 9). The results of migration analyses indicated the presence of biased migration, i.e., migration was higher from the Edmond field than from the other two vent fields (Figure 7). The egg of *Alviniconcha* sp. type 3 shows neutral buoyancy under atmospheric observation (Watanabe et al. unpublished data). Planktotrophic larval development is inferred from the protoconch morphology of *Alviniconcha* sp. type 3 [43], and enables potentially wide geographical dispersal and distribution. Gene flow of *Alviniconcha* gastropods was discussed previously [29], and was reported to differ between lineages, i.e., gene flow between the Manus Basin and the North Fiji Basin was attributed to *Alviniconch*a sp. type 1 and *Alviniconch*a sp. type 2; in contrast, gene flow of *Alviniconcha* sp. type 4 was limited to the Lau Basin. It appears that dispersal of this species is not constrained by horizontal distance, but by dispersal barriers that probably differ among species. Taken together, our results suggest that the Edmond population represents the potential source population for *Alviniconcha* sp. type 3 in the four vent fields, and that transform faults do not act as dispersal barriers for this species.

The scaly-foot gastropod was present in the Solitaire field and at a single chimney in the Kairei field. The average shell width in the Solitaire population was slightly smaller than that in the Kairei population [23]. The results of statistical analyses revealed no genetic differentiation between the two populations (Table 7), inferring potential connectivity between the two vent fields; however, the egg of the scaly-foot gastropod shows negative buoyancy under atmospheric pressure (Watanabe et al. unpublished data). Topological depressions were previously shown to act as dispersal barriers for polychaete species with negatively buoyant eggs [44]. The results of migration analyses indicated that the relative number of migrants per generation was higher from the Kairei field to the Solitaire field than from the Solitaire field to the Kairei field (Figure 7). Taken together, our results suggest that the Kairei population represents the potential source population for the two populations in the CIR. Recently, an additional scaly-foot assemblage was discovered in the Southwestern Indian Ridge [45]. Further studies, including investigations of this newly discovered population, will provide an insight into the dispersal ability and evolution of this unusual gastropod.

Potential dispersal barriers of vent-endemic animal species have mainly been reported for populations in the fast-spreading EPR, along which topological depression and interception of microplates appear to act as barriers for gene flow [9]. On the other hand, very little genetic differentiation has been reported for population in the slow-spreading MAR. The results of our present investigation of CIR vent-endemic animal species revealed almost no genetic differentiation among the four vent populations. Taken together, these findings indicate that the existence of relatively high connectivity among populations in slow- and intermediate- (<60 mm/year) spreading ridge systems. High variability in physical and chemical features of hydrothermal activities and fluids has been demonstrated for slow- and ultraslow-spreading ridge systems, such as the MAR and Mid-Cayman Rise [46]; this variability provides diversification of habitats and niches for animals associated with vent environments. Habitat diversity strongly affects the adaptation and connectivity of animals with various dispersal strategies. On the other hand, in arc-backarc systems of the western Pacific, in which vent-endemic animal communities are believed to form a single biogeographical province [18], [20], connectivity among vent populations is more complex than that occurring in mid-oceanic ridge systems; this complexity arises because arc-backarc systems are generally surrounded by island arcs, which prohibit dispersal of thermally intolerant larvae [47], [48].

In summary, the results of our present study provide an overview of the dispersal and population conditions of representative vent-associated fauna in four deep-sea hydrothermal vent fields along the CIR. Few previous investigations of the Indian Ocean Ridge system have been conducted; thus, our present findings not only provide new insights into larval dispersal and population establishment, but also help to clarify previously reported geochemical and biogeographical diversification [22], [23]. Additional biogeographical and ecophysiological studies of vent animals in the known and newly discovered vent fields of the CIR, and in other geographical and geological settings such as the East Scotia Ridge and Mid-Cayman Rise [46], [49], will further contribute to our understanding of larval dispersal and population establishment in global deep-sea chemosynthetic faunal communities.
